# Socioeconomic status and early blood concentrations of inflammation-related and neurotrophic proteins among extremely preterm newborns

**DOI:** 10.1371/journal.pone.0214154

**Published:** 2019-03-26

**Authors:** Alan Leviton, Elizabeth N. Allred, Olaf Dammann, Robert M. Joseph, Raina N. Fichorova, T. Michael O’Shea, Karl C. K. Kuban

**Affiliations:** 1 Boston Children’s Hospital and Harvard Medical School, Boston, MA, United States of America; 2 Tufts University School of Medicine, Boston, MA, United States of America; 3 Boston University School of Medicine, Boston, MA, United States of America; 4 Brigham and Women's Hospital and Harvard Medical School, Boston, MA, United States of America; 5 University of North Carolina School of Medicine, Chapel Hill, NC, United States of America; 6 Boston Medical Center and Boston University School of Medicine, Boston, MA, United States of America; Hopital Robert Debre, FRANCE

## Abstract

The main objective of this study was to evaluate the relationship between mother’s socioeconomic disadvantage and blood concentrations of inflammation-related proteins among extremely preterm newborns (<28 weeks gestation), a group at heightened risk of cognitive impairment when exposed to systemic inflammation. We measured the concentrations of 27 inflammatory and neurotrophic proteins in blood specimens collected a week apart during the first postnatal month from 857 extremely preterm newborns in the United States. We classified children according to 3 indicators/correlates of socioeconomic disadvantage, mother’s eligibility for government-provided medical care insurance (Medicaid), mother’s formal education level, and mother’s IQ approximated with the Kaufman Brief Intelligence Test– 2. The risks of a top-quartile concentration of each protein on each of 5 days a week apart, on two occasions during the first two postnatal weeks, and during the next two weeks were modeled as functions of each indicator of socioeconomic disadvantage. The risks of top quartile concentrations of multiple (2–5) inflammation-related proteins on multiple days during the first two weeks were increased for each of the 3 indicators of socioeconomic disadvantage, while the risks of top quartile concentrations of selected neurotrophic proteins were reduced. Adjustment for socioeconomic disadvantage did not alter the relationships between protein concentrations and both low IQ and low working memory 10 years later. Among extremely preterm newborns, indicators of socioeconomic disadvantage are associated with modestly increased risk of systemic inflammation in postnatal blood during the first postnatal month and with a slightly reduced risk of a neurotrophic signal, but do not confound relationships between protein concentrations and outcomes.

## 1. Introduction

Children born at term[1], and those born preterm [2] into families with socioeconomic disadvantages tend to have lower cognition scores than their peers born into families with socioeconomic advantages. Multiple explanations have been offered for this disparity. Most invoke allostatic load, the term applied to the stress that accompanies/follows from the need for constant adaptation[3].

Poor families face significant economic pressure and prejudice as they struggle to earn a living, pay bills, and make decisions about what is absolutely essential[4]. The excessively stressed parents who head these families may be less able than others to nurture, stimulate, and respond to their children's needs[5]. Such lower-quality parenting has been associated with the child’s limitations[6].

The biologic consequences of high levels of stress include effects on cortisol synthesis and metabolism[7], as well as epigenetic[8, 9] and pro-inflammatory[10–12] effects. These have been invoked to explain the relationships between socio-economic limitations and brain development[13]. In this report, we focus on inflammation-related proteins and neurotrophins, which appear capable of influencing the risks of brain damage associated with the adversities associated with socioeconomic disadvantage[14].

Previous reports of the relationship between socioeconomic disadvantage and inflammation in children have studied the gravida’s blood[11], the placenta[10], cord blood[10], infant’s saliva[12], and the blood of school-age children[15]. The biological embedding model of early adversity postulates that early adversity can become “embedded” in immune cells, resulting in a "proinflammatory phenotype" that persists for years and perhaps decades[16–18].

We could not find any report that assessed the relationship between socioeconomic disadvantage and inflammation during the weeks after extremely preterm birth. This gap in our knowledge is especially important in light of the evidence that socioeconomic disadvantage appears to influence brain development[19], and that early postnatal inflammation, especially if sustained, is associated with heightened risk of brain damage among children born months before term[20].

Data from the ELGAN (Extremely Low Gestational Age Newborn) Study provided an opportunity to fill this gap because of the availability of information about mother’s level of formal education, her eligibility for government-provided medical care insurance (Medicaid), and her score on the Kaufman Brief Intelligence Test[21], a brief, individually administered measure of verbal and nonverbal cognitive ability, as well as information about the concentrations of 27 proteins with inflammatory and/or neurotrophic properties measured in blood specimens obtained during the first postnatal month from their babies born before the 28^th^ week of gestation. The ELGAN Study also assessed IQ and working memory when the children were 10 years old, providing an opportunity to evaluate the effect of adjusting for socio-economic indicators when assessing the relationship between high newborn concentrations of inflammatory and neurotrophic proteins and low IQ 10 years later, as well as between high concentrations and limited working memory.

## Methods

### Participants

The ELGAN study is a multi-center prospective, observational study of the risk of structural and functional neurologic disorders in extremely preterm infants[22]. A total of 1506 infants born before the 28^th^ week of gestation were enrolled during the years 2002–2004. Of these infants, 857 provided a spot of blood on more than two occasions during the first postnatal month and returned for an assessment of IQ and working memory at age 10 years (Table 1) (all tables are in the results section). The ethics / human-subject-protection /institutional review / institutional care and use committees of Boston Children’s Hospital, Boston MA, Tufts Medical Center, Boston MA, University of Massachusetts Medical School, Worcester MA, Yale University School of Medicine, New Haven, CT, Wake Forest University Baptist Medical Center, Winston-Salem NC, University Health Systems of Eastern Carolina, Greenville, NC, North Carolina Children's Hospital, Chapel Hill, NC, Helen DeVos Children's Hospital, Grand Rapids, MI, Sparrow Hospital, Lansing, MI, University of Chicago Medical Center, Chicago, IL, and William Beaumont Hospital, Royal Oak, MI specifically approved this study.

**Table 1:** Odds ratios and 95% confidence intervals of a top quartile concentration of the protein listed on the left associated with mother’s eligibility for government-provided medical care insurance. The only adjustments made were for gestational age category and birth weight Z-score < -1. **Bold** indicates odds ratios significantly > 1.0 (p < 0.05) and ***bold italic*** indicates odds ratios significantly < 1.0 (p < 0.05).

	Day 1	Day 7	Day 14	Day 21	Day 28
CRP	1.0 (0.7, 1.4)	1.2 (0.9, 1.7)	1.0 (0.7, 1.4)	0.8 (0.5, 1.1)	0.9 (0.6, 1.3)
SAA	1.2 (0.8, 1.7)	1.4 (1.00, 1.9)	1.2 (0.9, 1.7)	0.7 (0.5, 1.1)	0.9 (0.6, 1.3)
MPO	1.1 (0.8, 1.5)	1.2 (0.9, 1.7)	0.9 (0.6, 1.3)	0.7 (0.5, 1.01)	0.8 (0.5, 1.2)
IL-1β	1.1 (0.8, 1.6)	1.0 (0.7, 1.4)	1.0 (0.7, 1.5)	1.0 (0.7, 1.4)	0.9 (0.6, 1.3)
IL-6	1.3 (0.9, 1.8)	1.3 (0.9 1.8)	1.2 (0.8, 1.7)	0.9 (0.6, 1.3)	0.9 (0.6, 1.4)
IL-6R	1.1 (0.8, 1.5)	1.0 (0.7, 1.4)	***0*.*7 (0*.*5*, *0*.*9)***	0.7 (0.5, 1.01)	0.8 (0.5, 1.2)
TNF-α	1.1 (0.8, 1.5)	1.3 (0.96, 1.8)	1.1 (0.8, 1.6)	0.9 (0.6, 1.2)	0.8 (0.6, 1.2)
TNF-R1	1.0 (0.7, 1.4)	1.1 (0.8, 1.5)	0.7 (0.5, 1.1)	0.8 (0.6, 1.2)	***0*.*7 (0*.*5*, *0*.*99)***
TNF-R2	1.2 (0.8, 1.6)	**1.7 (1.2, 2.4)**	1.1 (0.8, 1.5)	1.3 (0.9, 1.9)	1.0 (0.7, 1.5)
IL-8	1.1 (0.8, 1.5)	**1.4 (1.01, 1.9)**	1.3 (0.9, 1.9)	1.4 (0.9, 2.0)	1.3 (0.9, 1.8)
RANTES	1.2 (0.9, 1.7)	1.1 (0.8, 1.5)	0.7 (0.5, 1.1)	0.8 (0.5, 1.1)	0.9 (0.6, 1.3)
ICAM-1	1.1 (0.8, 1.6)	**1.4 (1.01, 1.9)**	1.2 (0.9, 1.7)	1.1 (0.8, 1.6)	1.0 (0.7, 1.5)
VCAM-1	1.1 (0.8, 1.5)	0.9 (0.7, 1.3)	0.8 (0.6, 1.1)	0.9 (0.6, 1.3)	***0*.*6 (0*.*4*, *0*.*9)***
MMP-9	1.1 (0.8, 1.6)	1.3 (0.96, 1.9)	0.9 (0.6, 1.3)	0.8 (0.5, 1.1)	0.9 (0.6, 1.3)
TSH	1.2 (0.8, 1.6)	**1.5 (1.1, 2.1)**	0.9 (0.7, 1.3)	1.0 (0.7, 1.4)	***0*.*6 (0*.*4*, *0*.*9)***
EPO	0.9 (0.6, 1.2)	**1.5 (1.05, 2.0)**	1.1 (0.8, 1.5)	1.2 (0.8, 1.7)	0.8 (0.5, 1.1)
NT-4	1.0 (0.7, 1.4)	0.9 (0.6, 1.3)	0.7 (0.5, 1.04)	0.8 (0.5, 1.1)	0.8 (0.6, 1.2)
BDNF	1.1 (0.8, 1.6)	0.9 (0.6, 1.3)	0.7 (0.5, 1.03)	0.7 (0.5, 1.01)	1.1 (0.7, 1.6)
bFGF	0.8 (0.6, 1.1)	0.8 (0.5, 1.1)	***0*.*6 (0*.*4*, *0*.*9)***	0.7 (0.5, 1.1)	***0*.*7 (0*.*5*, *0*.*99)***
IGF-1	***0*.*6 (0*.*4*, *0*.*9)***	0.8 (0.5, 1.1)	0.9 (0.6, 1.3)	0.9 (0.6, 1.3)	0.7 (0.5, 1.1)
IGFBP-1	1.0 (0.7, 1.4)	0.9 (0.7, 1.3)	1.1 (0.8, 1.5)	1.0 (0.7, 1.5)	0.9 (0.6, 1.3)
VEGF	1.0 (0.7, 1.4)	**1.4 (1.03, 2.0)**	0.9 (0.6, 1.3)	0.7 (0.5, 1.05)	0.7 (0.5, 1.04)
VEGF-R1	1.0 (0.7, 1.3)	0.8 (0.6, 1.1)	0.7 (0.5, 1.00)	1.1 (0.8, 1.6)	1.1 (0.7, 1.6)
VEGF-R2	1.3 (0.97, 1.9)	1.3 (0.97, 1.9)	0.9 (0.6, 1.3)	1.3 (0.9, 1.8)	0.8 (0.6, 1.2)
PIGF	0.8 (0.5, 1.1)	0.8 (0.5, 1.2)	***0*.*5 (0*.*3*, *0*.*7)***	0.9 (0.7, 1.3)	0.8 (0.5, 1.2)
Ang-1	1.3 (0.9, 1.8)	0.9 (0.6, 1.3)	***0*.*5 (0*.*3*, *0*.*8)***	***0*.*7 (0*.*5*, *0*.*97)***	0.9 (0.6, 1.3)
Ang-2	1.2 (0.8, 1.7)	1.0 (0.7, 1.4)	***0*.*6 (0*.*4*, *0*.*9)***	1.2 (0.8, 1.7)	1.0 (0.7, 1.5)
Total N	669	682	618	566	531

### Maternal characteristics

After delivery, a trained research nurse interviewed each mother in her native language following a structured procedure. The mothers were asked about their formal education, eligibility for government-provided medical care insurance (Medicaid), age, marital status, and racial and ethnic identity. Ten years later when they brought their child back for an assessment, 812 completed the Kaufman Brief Intelligence Test– 2 (KBIT-2)[21] nonverbal subscale. This assessment was initially intended to approximate the heritable component of the child’s IQ. However, here this information is offered as a correlate/surrogate of low socioeconomic (SES)[23].

### Systemic inflammation ascertainment procedures

Drops of blood were collected on filter paper on the first postnatal day (range: 1–3 days), the 7^th^ postnatal day (range: 5–8 days), the 14^th^ postnatal day (range: 12–15 days), the 21^st^ postnatal day (range: 19–23 days), and the 28^th^ postnatal day (range: 26–29). All blood was from the remainder of specimens obtained for clinical indications. Dried blood spots were stored at -70 ^O^C in sealed bags with a desiccant until processed. Details about the elution of proteins from the blood spots are provided elsewhere[24]. The total protein concentration in each eluted sample was determined by bicinchoninic acid (BCA) assay (Thermo Scientific, Rockford, IL) using a multi-label Victor 2 counter (Perkin Elmer, Boston, MA) and the measurements of each analyte normalized to mg total protein.

#### Proteins measured

Our choice of which proteins to measure was limited by financial constraints. Some of the proteins we did measure have since been shown to be key players in inflammation networks[25], neurotrophin networks[26], and in angiogenic networks[27].

The Genital Tract Biology Laboratory at the Brigham and Women’s Hospital in Boston Massachusetts eluted all blood spots as previously described and measured all proteins reported here. The laboratory used the Meso Scale Discovery to measure: C-Reactive Protein (CRP), SAA, Myeloperoxidase (MPO) Interleukin-1 β (IL-1β), IL-6, IL-6 Receptor (IL-6R), Tumor Necrosis Factor-α (TNF-α), TNF Receptor-1 (TNFR-1), TNFR-2, IL-8 (CXCL8), Regulated upon Activation, Normal T-cell Expressed, and Secreted (RANTES; CCL5), Intercellular Adhesion Molecule -1 (ICAM-1; CD54), Vascular Cell Adhesion Molecule-1 VCAM-1; CD106), Vascular Endothelial Growth Factor (VEGF), VEGF Receptor-1 (VEGFR-1, also known as sFLT-1), VEGFR-2 (KDR), Insulin-Like Growth Factor-1 (IGF) Binding Protein-1 (IGFBP-1), thyroid stimulating hormone (TSH), Metalloproteinase (MMP)-9, and erythropoietin (EPO).

A multiplex immunobead assay manufactured by R&D Systems (Minneapolis, MN) and a MAGPIX Luminex reader (R&D Systems) were used to measure angiopoietin-1 (Ang-1), Ang-2, placenta growth factor (PIGF), Neurotrophin-4 (NT-4), Brain Derived Neurotrophic Factor (BDNF), and basic Fibroblast Growth Factor (bFGF). ELISA (R&D Systems) was used to measure IGF-1.

The protein concentrations varied with gestational age, and with the postnatal day of collection[28, 29]. We created two epochs for two reasons. First, we measured protein concentrations in two separate sets with the “earlier” specimens (days 1, 7, and 14) measured in 2010, and the “later” specimens (days 21 and 28) measured in 2014–2015. Second, by the third post-natal week, the infants who no longer needed assisted ventilation were no longer having blood drawn for blood gas measurements. Consequently, the infants whose blood was measured after day 14 were a biased sample composed of the “sickest” infants. Although the distributions of protein concentrations in each epoch were similar, they were not identical. Because we were interested in the contribution of high concentrations, and the concentrations of most proteins did not follow a normal distribution, the distribution of each protein’s concentration was dichotomized into the highest quartile and the lower three quartiles among children in each of the 30 groups.

### Procedures at age 10 years

Only children for whom we had a blood specimen were invited to be evaluated. General cognitive ability (or IQ) was assessed with the School-Age Differential Ability Scales–II (DAS-II) Verbal and Nonverbal Reasoning scales[30]. Working memory was also assessed with the DAS-II[30] among the 692 of the 857 children who had a DAS-II mean IQ ≥ 70. Of these 692, 164 had a DAS-II working memory Z-score ≤ -1

### Data analyses

We evaluated the general null hypothesis that the risks of systemic inflammation, and of a neurotrophic signal, during the first postnatal month, were not associated with indicators of socioeconomic disadvantage. To accomplish these goals, we created logistic regression models that provided estimates of odds ratios and 95% confidence intervals of the risk of top-quartile concentrations of each protein after adjustment for gestational age category and birthweight Z-score < -1. Because protein concentrations among girls were very similar to those of boys, we did not adjust for the newborn’s sex.

We began our analysis with an assessment of the relationship between top-quartile concentrations of proteins on individual days and three maternal indicators of socioeconomic disadvantage, mother’s eligibility for government-provided medical care insurance (Medicaid)(Table 2), maternal education (≤ 16 years formal education)(S1 Table), and mother’s IQ < 85 (S2 Table). Because sustained or intermittent inflammation has been most clearly associated with increased risk of indicators of brain damage, we sought relationships between top-quartile concentrations of proteins on multiple days during what we call the early epoch (days 1, 7, and 14) (Table 3), and separately, the late epoch (days 21 and 28) (Table 4) and mother’s education (≤ 12 years, >12, <16 years). Then we explored if adjusting for mother’s socioeconomic disadvantage makes any difference when evaluating the relationship between a top quartile protein concentration on 2 days during the early epoch and the child’s risk of an IQ less than 70, and separately, among children whose IQ ≥ 70, the relationship between a top quartile protein concentration on 2 days during the early epoch and the child’s risk of a working memory score more than one standard deviation below the expected mean (Table 5 and S3 and S4 Tables). Finally, we did the same for top quartile protein concentrations on 2 days during the late epoch (S5 and S6 Tables)

**Table 2:** Odds ratios (95% confidence intervals) for a top-quartile concentration of the protein listed on the left on 2 days during the early epoch among children whose mother had the characteristic listed at the top of the column relative to the risk among children who did not have that characteristic. The referent group for the 2 maternal education characteristics is comprised of children whose mother had the equivalent of a college education. Adjustment is made for gestational age category and birth weight Z-score < -1. **Bold** indicates odds ratios significantly > 1 (p < 0.05) and ***bold italic*** indicates odds ratios significantly < 1 (p < 0.05).

	Mother’s education (years)		Medicaid	Mother’s
	≤ 12	>12, <16	eligible	KBIT < 85
CRP	1.1 (0.8, 1.7)	1.2 (0.8, 2.0)	1.0 (0.7, 1.4)	1.1 (0.7, 1.9)
SAA	**1.5 (1.01, 2.3)**	0.9 (0.6, 1.6)	1.4 (0.99, 2.1)	1.2 (0.7, 2.1)
MPO	0,8 (0.6, 1.4)	1.2 (0.8, 1.9)	1.3 (0.9, 1.8)	1.2 (0.7, 2.0)
IL-1β	0.9 (0.6, 1.4)	0.9 (0.6, 1.5)	0.9 (0.6, 1.4)	0.8 (0.5, 1.6)
IL-6	1.4 (0.9, 2.2)	1.3 (0.8, 2.2)	1.3 (0.9, 2.0)	**1.9 (1.1, 3.2)**
IL-6R	1.3 (0.9, 1.9)	1.0 (0.6, 1.6)	1.0 (0.7, 1.4)	**1.7 (1.1, 2.9)**
TNF-α	1.5 (0.97, 2.3)	**2.1 (1.3, 3.3)**	**1.5 (1.04, 2.2)**	1.4 (0.8, 2.4)
TNF-R1	1.1 (0.7, 1.6)	1.4 (0.8, 2.2)	0.9 (0.6, 1.3)	0.8 (0.5, 1.5)
TNF-R2	1.3 (0.9, 2.1)	**2.2 (1.4, 3.6)**	**1.5 (1.02, 2.1)**	1.4 (0.8, 2.3)
IL-8	1.4 (0.9, 2.3)	**2.1 (1.3, 3.3)**	**1.6 (1.1, 2.2)**	1.0 (0.6, 1.8)
RANTES	1.3 (0.9, 1.9)	1.3 (0.8, 2.0)	1.1 (0.8, 1.6)	1.5 (0.9, 2.5)
ICAM-1	1.4 (0.9, 2.1)	1.5 (0.9, 2.4)	1.4 (0.97, 2.0)	1.2 (0.7, 2.1)
VCAM-1	1.2 (0.8, 1.8)	1.0 (0.6, 1.6)	0.9 (0.6, 1.2)	1.4 (0.8, 2.4)
MMP-9	1.0 (0.6, 1.5)	1.3 (0.8, 2.2)	1.2 (0.8, 1.8)	1.0 (0.5, 1.8)
TSH	**1.6 (1.1, 2.3)**	1.0 (0.6, 1.6)	**1.5 (1.04, 2.1)**	1.2 (0.7, 2.0)
EPO	1.4 (0.9, 2.2)	1.4 (0.9, 2.3)	1.3 (0.9, 1.8)	1.0 (0.5, 1.7)
NT-4	1.0 (0.7, 1.5)	0.8 (0.5, 1.3)	0.9 (0.7, 1.4)	0.9 (0.5, 1.6)
BDNF	1.3 (0.9, 2.0)	1.1 (0.7, 1.8)	0.9 (0.6, 1.3)	1.5 (0.9, 2.5)
bFGF	0.7 (0.5, 1.1)	0.9 (0.5, 1.4)	0.7 (0.5, 1.04)	0.8 (0.4, 1.6)
IGF-1	0.9 (0.6, 1.4)	0.9 (0.6, 1.5)	0.8 (0.5, 1.1)	0.8 (0.5, 1.6)
IGFBP-1	1.0 (0.7, 1.6)	1.1 (0.7, 1.8)	1.1 (0.7, 1.6)	0.9 (0.5, 1.7)
VEGF	1.1 (0.7, 1.7)	**1.7 (1.1, 2.7)**	1.1 (0.8, 1.6)	0.8 (0.5, 1.5)
VEGF-R1	***0*.*6 (0*.*4*, *0*.*9)***	0.9 (0.6, 1.4)	***0*.*6 (0*.*4*, *0*.*8)***	***0*.*4 (0*.*1*, *0*.*9)***
VEGF-R2	**1.5 (1.02, 2.3)**	1.5 (0.9, 2.3)	1.3 (0.9, 1.8)	0.9 (0.5, 1.6)
PIGF	0.7 (0.5, 1.1)	0.6 (0.4, 1.1)	0.7 (0.4, 1.01)	0.6 (0.3, 1.3)
Ang-1	1.1 (0.8, 1.7)	1.0 (0.6, 1.6)	0.8 (0.6, 1.2)	1.5 (0.8, 2.1)
Ang-2	1.1 (0.7, 1.7)	1.3 (0.8, 2.2)	0.8 (0.6, 1.2)	0.7 (0.4, 1.4)

**Table 3:** Odds ratios (95% confidence intervals) for a top-quartile concentration of the protein listed on the left on 2 days during the late epoch among children whose mother had the characteristic listed at the top of the column relative to the risk among children who did not have that characteristic. The referent group for the 2 maternal education characteristics is comprised of children whose mother had the equivalent of a college education. **Bold** indicates odds ratios significantly > 1 (p < 0.05) and ***bold italic*** indicates odds ratios significantly < 1 (p < 0.05).

	Mother’s education (years)		Medicaid	Mother’s
	≤ 12	>12, <16	eligible	KBIT < 85
CRP	1.2 (0.6, 2.1)	0.7 (0.3, 1.5)	1.0 (0.6, 1.8)	0.6 (0.2, 1.7)
SAA	0.6 (0.3, 1.2)	0.6 (0.3, 1.3)	***0*.*5 (0*.*2*, *0*.*9)***	0.6 (0.2, 1.8)
MPO	0.9 (0.5, 1.6)	0.8 (0.4, 1.5)	0.8 (0.4, 1.3)	0.3 (0.1, 1.1)
IL-1β	1.5 (0.8, 2.8)	1.1 (0.5, 2.2)	0.8 (0.5, 1.5)	0.8 (0.3, 1.9)
IL-6	1.0 (0.6, 1.9)	0.6 (0.3, 1.3)	0.7 (0.4, 1.3)	1.7 (0.9, 3.5)
IL-6R	1.4 (0.7, 2.6)	**2.0 (1.02, 3.9)**	1.0 (0.6, 1.6)	1.0 (0.5, 2.2)
TNF-α	0.8 (0.5, 1.3)	0.7 (0.4, 1.3)	0.9 (0.6, 1.5)	1.3 (0.7, 2.5)
TNF-R1	0.7 (0.4, 1.4)	1.2 (0.6, 2.3)	0.7 (0.4, 1.3)	0.5 (0.2, 1.5)
TNF-R2	1.4 (0.7, 2.8)	**2.9 (1.5, 5.7)**	1.2 (0.7, 2.1)	1.1 0.5, 2.4)
IL-8	**2.0 (1.1, 3.5)**	1.4 (0.7, 2.8)	1.2 (0.7, 1.9)	**2.3 (1.3, 4.3)**
RANTES	1.0 (0.5, 1.8)	1.1 (0.5, 2.1)	0.9 (0.5, 1.6)	0.6 (0.2, 1.6)
ICAM-1	**1.8 (1.02, 3.1)**	1.6 (0.8, 3.0)	0.8 (0.5, 1.4)	1.7 (0.9, 3.1)
VCAM-1	0.8 (0.4, 1.5)	1.1 (0.6, 2.2)	***0*.*5 (0*.*3*, *0*.*95)***	0.9 (0.4, 2.0)
MMP-9	1.4 (0.7, 2.8)	1.0 (0.4, 2.2)	1.1 (0.6, 2.0)	0.9 (0.4, 2.3)
TSH	0.6 (0.4, 1.1)	0.8 (0.4, 1.5)	0.8 (0.5, 1.3)	1.1 (0.5, 2.2)
EPO	0.6 (0.3, 1.1)	0.6 (0.3, 1.1)	1.1 (0.7, 1.9)	0.9 (0.4, 2.0)
NT-4	0.6 (0.3, 1.1)	0.9 (0.5, 1.8)	0.7 (0.4, 1.2)	0.7 (0.3, 1.7)
BDNF	0.7 (0.4, 1.3)	0.7 (0.4, 1.30	0.8 (0.5, 1.3)	0.7 (0.3, 1.7)
bFGF	***0*.*5 (0*.*3*, *0*.*9)***	0.6 (0.3, 1.1)	***0*.*4 (0*.*3*, *0*.*8)***	——
IGF-1	0.8 (0.5, 1.4)	0.8 (0.5, 1.5)	0.9 (0.5, 1.4)	1.1 (0.5, 2.1)
IGFBP-1	1.2 (0.6, 2.2)	0.9 (0.4, 1.9)	0.8 (0.5, 1.5)	0.8 (0.3, 2.1)
VEGF	0.8 (0.4, 1.3)	***0*.*4 (0*.*2*, *0*.*9)***	0.7 (0.4, 1.2)	0.4 (0.1, 1.2)
VEGF-R1	1.0 (0.6, 1.9)	0.7 (0.3, 1.4)	1.3 (0.8, 2.2)	0.7 (0.3, 1.8)
VEGF-R2	1.5 (0.8, 2.8)	1.8 (0.97, 3.7)	1.2 (0.7, 1.9)	1.2 (0.6, 2.5)
PIGF	0.6 (0.3, 1.1)	0.6 (0.3, 1.2)	0.5 (0.3, 1.00)	***0*.*2 (0*.*01*, *0*.*9)***
Ang-1	0.7 (0.4, 1.2)	0.5 (0.3, 1.03)	0.6 (0.4, 1.04)	0.9 (0.4, 1.9)
Ang-2	1.2 (0.7, 2.0)	1.4 (0.8, 2.6)	1.1 (0.7, 1.8)	1.4 (0.7, 2.8)

**Table 4:** Odds ratios (95% confidence intervals) for the 10-year old having an IQ less than 70, and separately, among children whose IQ ≥ 70, having a working memory more than one standard deviation below the expected mean associated with a top-quartile concentration of the protein listed on the left on 2 days during the early epoch. In one set of analyses adjustment is made for mother’s Medicaid eligibility at the time of delivery, while in another, such adjustment is not made although in both situations adjustment is made for gestational age category and birth weight Z-score < -1. **Bold** indicates odds ratios significantly > 1 (p < 0.05) and ***bold italic*** indicates odds ratios significantly < 1 (p < 0.05).

				Working memory Z-score ≤ -1	
		IQ < 70		when IQ ≥ 70	
	Medicaid	Adjusted for Medicaid eligibility		Adjusted for Medicaid eligibility	
	eligible *	No	Yes	No	Yes
CRP	1.0 (0.7, 1.4)	**1.7 (1.1, 2.7)**	**1.8 (1.1, 2.9)**	1.3 (0.8, 2.0)	1.3 (0.8, 2.1)
SAA	1.4 (0.99, 2.1)	1.4 (0.9, 2.4)	1.4 (0.8, 2.3)	1.1 (0.7, 1.8)	1.0 (0.6, 1.7)
MPO	1.3 (0.9, 1.8)	1.0 (0.6, 1.7)	0.9 (0.5, 1.6)	0.8 (0.5, 1.3)	0.8 (0.5, 1.3)
IL-1β	0.9 (0.6, 1.4)	1.4 (0.9, 2.4)	1.5 (0.9, 2.5)	1.1 (0.7, 1.8)	1.1 (0.7, 1.8)
IL-6	1.3 (0.9, 2.0)	**2.0 (1.2, 3.3)**	**1.9 (1.2, 3.2)**	1.3 (0.8, 2.0)	1.2 (0.7, 1.9)
IL-6R	1.0 (0.7, 1.4)	0.6 (0.3, 1.1)	0.6 (0.3. 1.1)	0.9 (0.6, 1.4)	0.9 (0.6, 1.4)
TNF-α	**1.5 (1.04, 2.2)**	**1.8 (1.1, 2.8)**	1.6 (1.00, 2.7)	**2.1 (1.4, 3.2)**	**2.0 (1.3, 3.1)**
TNF-R1	0.9 (0.6, 1.3)	1.3 (0.8, 2.1)	1.3 (0.8, 2.3)	1.2 (0.8, 1.9)	1.2 (0.8, 2.0)
TNF-R2	**1.5 (1.02, 2.1)**	1.3 (0.8, 2.2)	1.2 (0.7, 2.1)	1.1 (0.7, 1.8)	1.0 (0.6, 1.7)
IL-8	**1.6 (1.1, 2.2)**	**2.2 (1.3, 3.6)**	**2.1 (1.2, 3.4)**	**1.6 (1.03, 2.6)**	1.5 (0.96, 2.4)
RANTES	1.1 (0.8, 1.6)	0.8 (0.5, 1.4)	0.7 (0.4, 1.3)	0.9 (0.6, 1.4)	0.9 (0.6, 1.5)
ICAM-1	1.4 (0.97, 2.0)	**2.3 (1.5, 3.7)**	**2.2 (1.4, 3.6)**	1.4 (0.9, 2.3)	1.5 (0.9, 2.3)
VCAM-1	0.9 (0.6, 1.2)	1.3 (0.8, 2.1)	1.3 (0.8, 2.2)	1.1 (0.7, 1.7)	1.2 (0.7, 1.8)
MMP-9	1.2 (0.8, 1.8)	0,7 (0.4, 1.3)	0.6 (0.3, 1.2)	1.1 (0.7, 1.8)	1.1 (0.7, 1.8)
TSH	**1.5 (1.04, 2.1)**	1.1 (0.7, 1.8)	0.9 (0.6, 1.6)	1.1 (0.7, 1.7)	1.0 (0.7, 1.6)
EPO	1.3 (0.9, 1.8)	**1.9 (1.2, 3.1)**	**1.9 (1.1, 3.1)**	**1.6 (1.04, 2.6)**	**1.6 (1.02, 2.5)**
NT-4	0.9 (0.7, 1.4)	1.3 (0.8, 2.1)	1.3 (0.8, 2.2)	1.3 (0.8, 2.0)	1.3 (0.8, 2.0)
BDNF	0.9 (0.6, 1.3)	0.7 (0.4, 1.3)	0.7 (0.4, 1.3)	0.9 (0.6, 1.4)	0.9 (0.6, 1.4)
bFGF	0.7 (0.5, 1.04)	1.1 (0.6, 1.9)	1.2 (0.7, 2.1)	1.0 (0.6, 1.6)	1.1 (0.7, 1.8)
IGF-1	0.8 (0.5, 1.1)	0.9 (0.5, 1.6)	1.0 (0.6, 1.8)	0.7 (0.4, 1.2)	0.7 (0.4, 1.2)
IGFBP-1	1.1 (0.7, 1.6)	1.2 (0.7, 2.0)	1.2 (0.7, 2.0)	1.1 (0.7, 1.7)	1.1 (0.6, 1.7)
VEGF	1.1 (0.8, 1.6)	0.7 (0.4, 1.2)	0.7 (0.4, 1.2)	1.0 (0.6, 1.5)	0.9 (0.6, 1.4)
VEGF-R1	***0*.*6 (0*.*4*, *0*.*8)***	0.8 (0.4, 1.3)	0.9 (0.5, 1.6)	1.0 (0.6, 1.6)	1.1 (0.7, 1.7)
VEGF-R2	1.3 (0.9, 1.8)	1.2 (0.8, 2.0)	1.2 (0.7, 1.9)	1.2 (0.8, 1.8)	1.1 (0.7, 1.8)
PIGF	0.7 (0.4, 1.01)	0.7 (0.4, 1.4)	0.8 (0.4, 1.5)	1.1 (0.7, 1.8)	1.2 (0.7, 1.9)
Ang-1	0.8 (0.6, 1.2)	0.6 (0.3, 1.2)	0.7 (0.3, 1.2)	0.7 (0.4, 1.1)	0.7 (0.4, 1.1)
Ang-2	0.8 (0.6, 1.2)	1.2 (0.7, 2.1)	1.3 (0.8, 2.3)	1.1 (0.7, 1.8)	1.2 (0.7, 1.9)

* Repeated from Table 3

**Table 5:** Odds ratios (95% confidence intervals) for the 10-year old having an IQ less than 70, and separately, among children whose IQ ≥ 70, having a working memory more than one standard deviation below the expected mean associated with a top-quartile concentration of the protein listed on the left on 2 days during the late epoch. In one set of analyses adjustment is made for mother’s Medicaid eligibility at the time of delivery, while in another, such adjustment is not made although in both situations adjustment is made for gestational age category and birth weight Z-score < -1. **Bold** indicates odds ratios significantly > 1 (p < 0.05) and ***bold italic*** indicates odds ratios significantly < 1 (p < 0.05).

				Working memory Z-score ≤ -1	
		IQ < 70		when IQ ≥ 70	
	Medicaid	Adjusted for Medicaid eligibility		Adjusted for Medicaid eligibility	
	eligible *	No	Yes	No	Yes
CRP	1.0 (0.6, 1.8)	**2.0 (1.03, 3.7)**	**2.0 (1.02, 3.8)**	1.5 (0.8, 2.9)	1.5 (0.8, 2.9)
SAA	***0*.*5 (0*.*2*, *0*.*9)***	1.9 (0.9, 3.0)	**2.4 (1.1, 5.2)**	1.3 (0.6, 2.7)	1.3 (0.6, 2.9)
MPO	0.8 (0.4, 1.3)	0.8 (0.4, 1.7)	0.8 (0.4, 1.8)	1.3 (0.7, 2.3)	1.4 (0.7, 2.6)
IL-1β	0.8 (0.5, 1.5)	1.0 (0.5, 2.1)	1.1 (0.5, 2.2)	1.2 (0.6, 2.2)	1.2 (0.6, 2.3)
IL-6	0.7 (0.4, 1.3)	1.8 (0.96, 3.5)	**2.1 (1.1, 4.0)**	1.5 (0.8, 2.8)	1.6 (0.9, 3.1)
IL-6R	1.0 (0.6, 1.6)	0.9 (0.4, 1.8)	0.9 (0.4, 1.9)	0.9 (0.5, 1.7)	0.9 (0.5, 1.8)
TNF-α	0.9 (0.6, 1.5)	1.6 (0.9, 2.8)	1.6 (0.9, 3.0)	1.0 (0.6, 1.8)	1.0 (0.6, 1.8)
TNF-R1	0.7 (0.4, 1.3)	1.1 (0.5, 2.3)	1.2 (0.5, 2.6)	0.7 (0.3, 1.5)	0.7 (0.3, 1.6)
TNF-R2	1.2 (0.7, 2.1)	1.2 (0.6, 2.3)	1.2 (0.6, 2.3)	1.3 (0.7, 2.4)	1.3 (0.7, 2.4)
IL-8	1.2 (0.7, 1.9)	**2.7 (1.5, 4.8)**	**2.8 (1.5, 5.0)**	**2.1 (1.1, 3.8)**	**2.1 (1.1, 3.9)**
RANTES	0.9 (0.5, 1.6)	1.6 (0.9, 3.1)	1.6 (0.8, 3.2)	0.6 (0.3, 1.2)	0.6 (0.3, 1.2)
ICAM-1	0.8 (0.5, 1.4)	**2.4 (1.3, 4.2)**	**2.7 (1.5, 4.8)**	**1.8 (1.01, 3.2)**	**2.0 (1.1, 3.7)**
VCAM-1	***0*.*5 (0*.*3*, *0*.*95)***	0.9 (0.4, 1.8)	1.0 (0.5, 2.2)	0.8 (0.4, 1.5)	0.9 (0.4, 1.7)
MMP-9	1.1 (0.6, 2.0)	1.2 (0.6, 2.5)	1.2 (0.5, 2.5)	0.9 (0.4, 1.9)	0.9 (0.4, 2.0)
TSH	0.8 (0.5, 1.3)	**1.9 (1.1, 3.5)**	**2.1 (1.2, 4.0)**	0.8 (0.4, 1.5)	0.8 (0.4, 1.6)
EPO	1.1 (0.7, 1.9)	1.1 (0.6, 2.2)	1.1 (0.6, 2.2)	0.9 (0.5, 1.8)	0.9 (0.5, 1.8)
NT-4	0.7 (0.4, 1.2)	1.5 (0.8, 2.9)	1.7 (0.9, 3.3)	1.1 (0.6, 2.2)	1.2 (0.6, 2.3)
BDNF	0.8 (0.5, 1.3)	0.8 (0.4, 1.7)	0.8 (0.4, 1.8)	0.6 (0.3, 1.2)	0.6 (0.3, 1.3)
bFGF	***0*.*4 (0*.*3*, *0*.*8)***	0.9 (0.4, 1.9)	1.1 (0.5, 2.4)	0.8 (0.4, 1.6)	0.8 (0.4, 1.7)
IGF-1	0.9 (0.5, 1.4)	***0*.*4 (0*.*2*, *0*.*9)***	***0*.*4 (0*.*2*, *0*.*9)***	1.0 (0.6, 1.8)	1.0 (0.6, 1.8)
IGFBP-1	0.8 (0.5, 1.5)	**2.1 (1.01, 4.2)**	**2.2 (1.1, 4.8)**	1.0 (0.5, 2.2)	1.1 (0.5, 2.4)
VEGF	0.7 (0.4, 1.2)	0.9 (0.5, 1.9)	1.0 (0.5, 2.1)	1.1 (0.6, 2.0)	1.2 (0.6, 2.2)
VEGF-R1	1.3 (0.8, 2.2)	0.6 (0.3, 1.4)	0.6 (0.2, 1.4)	1.5 (0.8, 2.7)	1.4 (0.7, 2.5)
VEGF-R2	1.2 (0.7, 1.9)	1.7 (0.9, 3.2)	1.7 (0.9, 3.3)	1.2 (0.6, 2.3)	1.2 (0.7, 2.3)
PIGF	0.5 (0.3, 1.00)	0.8 (0.3, 1.9)	0.9 (0.4, 2.2)	0.6 (0.3, 1.3)	0.7 (0.3, 1.5)
Ang-1	0.6 (0.4, 1.04)	0.7 (0.3, 1.6)	0.8 (0.4, 1.7)	0.7 (0.4, 1.3)	0.7 (0.4, 1.4)
Ang-2	1.1 (0.7, 1.8)	**1.9 (1.1, 3.3)**	**1.9 (1.05, 3.3)**	0.9 (0.5, 1.6)	0.9 (0.5, 1.7)

* Repeated from Table 4

## Results

### Sample description (Fig 1)

The 857 children represented a wide socioeconomic range with 41% (N = 353) born to a mother who did not have formal education beyond high school, and 35% (N = 301) born to a mother who had completed a college program. 35% (N = 304) of the mothers were eligible for government-provided medical care insurance (Medicaid), while only 11% (N = 93) of mothers had an IQ approximation (KBIT-2 score) of less than 85. The three indicators of socio-economic disadvantage do not cluster uniformly. Of the 812 mothers who had all 3 assessments, 291 were Medicaid eligible, 526 had less than 16 years formal education and only 93 had a KBIT-2 score less than 85. Of those who were Medicaid eligible, fully 95% had less than 16 years formal education, yet only 19% had a KBIT-2 score less than 85. Of those who had less than 16 years formal education, 53% were Medicaid eligible, while only 14% had a KBIT-2 score less than 85. Fully 90% who had a low KBIT-2 score also had less than 16 years formal education, and 58% were Medicaid eligible.

**Fig 1:**
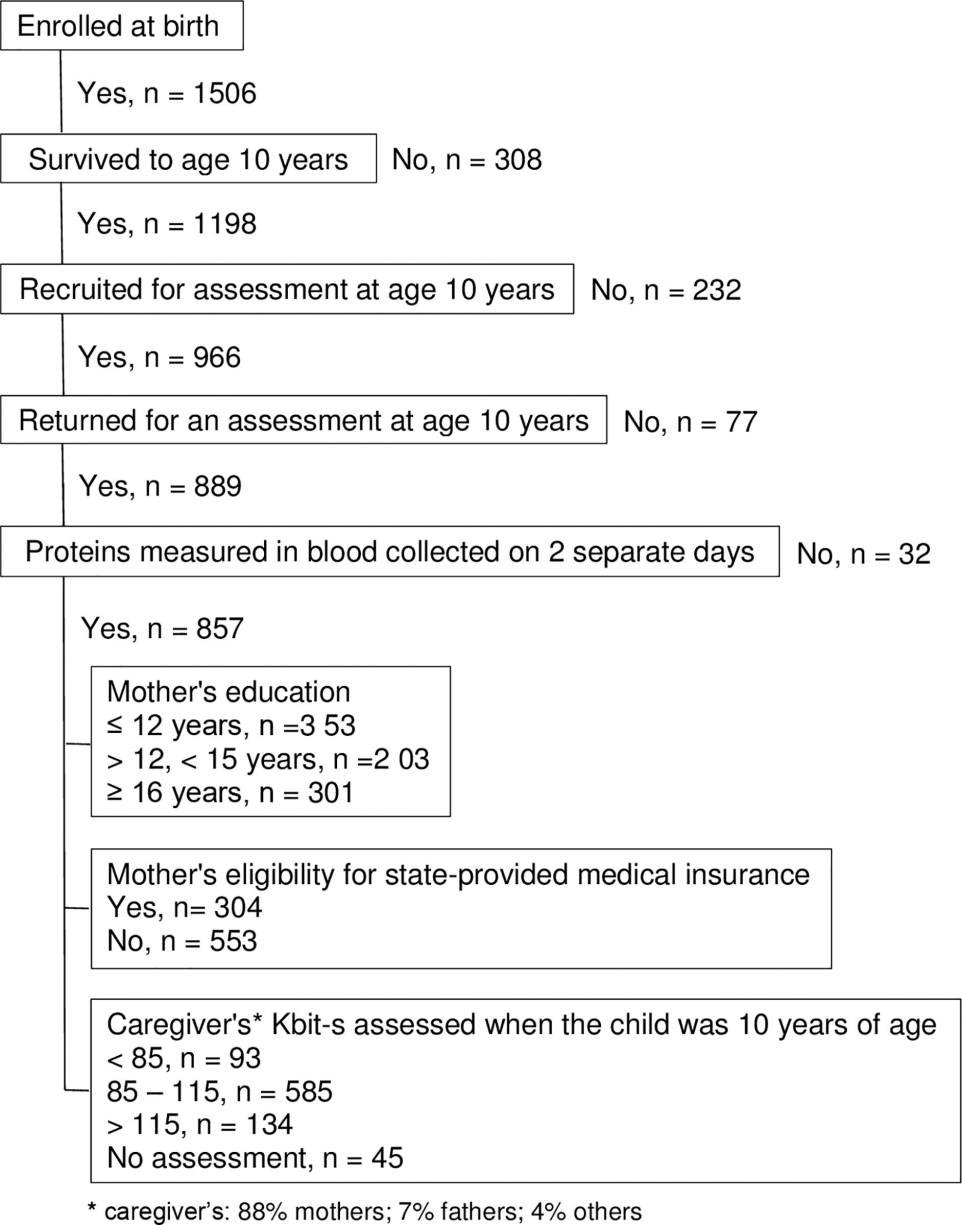


### Individual days (Table 1 and S1 and S2 Tables)

Children whose mother was eligible for Medicaid were at increased risk of having top-quartile day-7 concentrations of TNF-R2, IL-8, ICAM-1, TSH, EPO, and VEGF. (Table 2) In contrast, they were at reduced risk of top-quartile concentrations of proteins with neurotrophic properties, such as day-1 IGF-1, day-14 IL-6R, bFGF, PIGF, Ang-1,and Ang-2, day-21 Ang-1, and day-28 TNF-R1, VCAM-1, TSH, and bFGF.

Children whose mother had less than 16 years formal education was eligible for Medicaid were at increased risk of having top-quartile day-7 concentrations of CRP, SAA, IL-6, TNF-α, TNF-R2, ICAM-1, TSH, and VEGF-R2, as well as top-quartile day-14 concentrations of IL-6, TNF-R2, IL-8, ICAM-1, and VEGF-R2 (S1 Table). In addition, they were at reduced risk of top-quartile concentrations of proteins with neurotrophic properties, such as day-1 PIGF, day-14 NT-4, VEGFR-1, and Ang-1, day-28 TSH. When mother’s IQ (KBIT-2) < 85 is the indicator of socioeconomic disadvantage, inflammatory and neurotrophic signals are much less apparent than when other socioeconomic disadvantage indicators are the focus (S2 Table).

### Multiple days–early epoch (Table 2)

The concentrations of SAA, TSH, and VEGF-R2 were significantly higher among the children of mothers who had no formal education beyond high school, while the concentrations of TNF-α, TNF-R2, IL-8, and VEGF were significantly higher in the children of mothers who had some formal education beyond high school, but had not been graduated from college. Only the risk of a top-quartile concentration of VEGF-R1 was significantly lower among children of mothers who had no more than a high school education.

The children of mothers who were Medicaid eligible were appreciably more likely than others to have top-quartile concentrations of TNF-α, TNF-R2, IL-8, and TSH, and appreciably less likely to have a top-quartile concentration of VEGF-R1. Children of mothers whose KBIT was < 85 were at increased risk of top-quartile concentrations of IL-6 and IL-6R, and decreased risk of high concentrations of VEGF-R1.

### Multiple days–late epoch (Table 3)

The concentrations of IL-8 and ICAM-1 were significantly higher among the children of mothers who had no formal education beyond high school, while the concentrations of IL-6R and TNF-R2 were significantly higher in the children of mothers who had some formal education beyond high school, but had not been graduated from college. In contrast, the risk of a top-quartile concentration of bFGF was significantly lower among children of mothers who had no more than a high school education, and the risk of a top-quartile concentration of VEGF was significantly lower among children of mothers who had some formal education beyond high school, but had not been graduated from college.

The children whose mother was Medicaid eligible were not at increased risk of a top-quartile concentration of any protein. They were, however, at significantly reduced risk of top-quartile concentrations of SAA, VCAM-1, and bFGF.

Children of mothers whose KBIT was < 85 were at increased risk of a top-quartile concentration of IL-8 only, and at decreased risk of a high concentration of PIGF.

### Does adjustment for medicaid eligibility modify inflammation-associated or neurotrophin-associated early epoch risks of brain dysfunctions? (Table 4 and S3 and S4 Tables)

In our publications about the risks of brain structure and function abnormalities associated with systemic inflammation, we have not adjusted for indicators of social class because of our finding minimal relationship with late-epoch protein concentrations[31]. In light of what we found in Tables 2 and 3 and S1 and S2 Tables, however, we considered it prudent to explore the possibility that this strategy might have been sub-optimal.

The first column of Table 4 is repeated from Table 2 to show the relationship between Medicaid eligibility and the risk of elevated protein concentrations during the first epoch (*i*.*e*., first two postnatal weeks. By and large, adjusting for Medicaid eligibility has only trivial effects on the risks of an IQ < 70 (compare odds ratios in the second and third data columns), and of a low working memory score among children whose IQ ≥ 70 (compare odds ratios in the fourth and fifth data columns). Adjusting for mother’s limited education (<16 years) (S3 Table) and mother’s KBIT < 85 (S4 Table) also had only trivial effects on the inflammation-associated risks of an IQ < 70, and of a low working memory score among children whose IQ ≥ 70.

The first column is repeated from Table 3 to show the relationship between Medicaid eligibility and the risk of elevated protein concentrations during the first epoch (*i*.*e*., first two postnatal weeks (Table 4). By and large, adjusting for Medicaid eligibility has only trivial effects on the risks of an IQ < 70, and of a low working memory score among children whose IQ ≥ 70. Adjusting for mother’s limited education (<16 years) (S3 Table) and mother’s KBIT < 85 (S4 Table) also had only trivial effects on the risks of an IQ < 70, and of a low working memory score among children whose IQ was ≥ 70.

### Does adjustment for medicaid eligibility modify inflammation-associated or neurotrophin-associated late epoch risks of brain dysfunctions? (Table 5 and S5 and S6 Tables)

Table 5 is about the late epoch (i.e., the third and fourth postnatal weeks) equivalent of Table 4. The first column is repeated from Table 3 to show the relationship between Medicaid eligibility and the risk of elevated protein concentrations during the late epoch (*i*.*e*., the third and fourth postnatal weeks). Here, too, adjusting for Medicaid eligibility has only trivial effects on the risks of an IQ < 70, and of a low working memory score among children whose IQ ≥ 70. Adjusting for mother’s limited education (<16 years) (S5 Table) and mother’s KBIT < 85 (S6 Table) also had only trivial effects on the risks of an IQ < 70, and of a low working memory score among children whose IQ was ≥ 70.

## Discussion

From a broad, if over-simplified perspective, extremely preterm infants born to women at socioeconomic disadvantage are at a modestly increased risk of an inflammatory signal in postnatal blood during the first postnatal month, and slightly reduced risk of a neurotrophic signal. Our finding an association between indicators of mother’s socioeconomic disadvantage and increased risk of systemic inflammation during the first postnatal month in infants born extremely preterm is new. Nevertheless, it is in keeping with a literature that has documented relationships between socioeconomic disadvantage (and its correlates) and inflammation throughout the life span[9, 12, 15, 32–35].

Our finding an association between indicators of mother’s socioeconomic disadvantage and reduced availability of high concentrations of neurotrophic proteins among infants born extremely preterm is also new. Because neurotrophic proteins promote the survival and differentiation of the brain cells during embryonic and early postnatal stages[36], and an abundance of neurotrophic proteins is associated with reduced risks of brain damage[14], the reduced availability of high concentrations of neurotrophic proteins we found associated with socioeconomic disadvantage adds another possible explanation for the vulnerability of socioeconomically disadvantaged newborns.

The systemic inflammatory signal we found associated with indicators of socioeconomic disadvantage was limited to a few proteins (*e*.*g*., TNF-α or one of its receptors, IL-8, and ICAM-1) on a few days (especially day 7) (Table 2). Similarly, the “paucity of neurotrophin/angiogenin” profile was also limited in scope and time (especially to bFGF, PIGF, Ang-1, and Ang-2 on day 14). The importance of most of these proteins is evident in Tables 5 and 6, which show associations of high concentrations of some of the inflammation-related proteins with two indicators of brain damage/dysfunction. The importance of the “paucity of neurotrophin/angiogenin” profile is most evident in our publications that assessed the contributions of high concentrations of inflammation-related proteins in light of the concentrations of proteins with neurotrophic properties[37–41].

### Explanations for what we found

A number of studies view allostatic load with its resulting alterations in cortisol metabolism as perhaps the main biologic mechanism that explains health and other adversities.[42] In the ELGAN Study cohort, higher corticotropin-releasing hormone expression in the placenta was not associated with an inflammation profile in the newborn. Consequently, we are reluctant to invoke stress or altered cortisol metabolism as explanations for what we found.

In the ELGAN Study cohort, Medicaid eligibility was associated with recovery of a multiple organisms from placenta parenchyma, inflammation of the chorionic plate of the placenta and the base of the umbilical cord, and with recovery of bacteria from the newborn’s blood during the third and/or fourth postnatal weeks (unpublished observations). These observations raise the possibility that the correlates of socioeconomic disadvantage, rather than allostatic load and stress, might account for the associations maternal socioeconomic disadvantage has with inflammation and neurotrophin profiles in the newborn.

### Socioeconomic disadvantage

Some have created constructs for families’ level of function despite socioeconomic disadvantage that emphasize family stress[43], the costs of constant adaptation[3], and the resilience[44]. We are unable to incorporate these constructs in our analyses. Consequently, we classified newborns only by indicators of their mother’s socioeconomic disadvantage.

The three indicators, Medicaid eligibility, education level, and KBIT-2 score measure different aspects/correlates of socioeconomic disadvantage. Indicators of family income (such as Medicaid eligibility) appear to convey the most information about the risks of health,[45] and developmental[46–48] limitations, as well as educational opportunities[49] and educational achievement[4]. We found that newborns whose mother was Medicaid eligible or had a relatively low educational level had the most obvious, though limited, inflammatory signal and diminished neurotrophic signal during the first two postnatal weeks. This was in contrast to the less-evident signals among infants born to mothers with relatively low KBIT-2 scores.

Our finding that women who have a low KBIT-2 are less likely than other low SES women to give birth to an ELGAN with some systemic inflammation profile raises the possibility that either these women experience lower allostatic loads than their low SES peers or that the KBIT-2 is not a good indicator of allostatic load. We could not find any reports of allostatic load associated with low maternal IQ.

During the first 2 weeks, infants born to women who had some post-high-school education, but no post-college education were at increased risk of top-quartile concentrations of pro-inflammatory proteins as were infants born to women who had no formal education beyond high school (Table 3). The referent group for these analyses consists of infants whose mother had post-college education (including those with post-graduate degrees). These observations raise the possibility that in our highly economically-divided society, socioeconomic disadvantage is not limited to the lower extremes only.

### Individual proteins are surrogates for many other proteins

We view each protein as a surrogate for other proteins with similar capabilities or in the same or related pathways. We prefer not to focus on the identified inflammatory signals for TNF-α, IL-8, and ICAM-1, but rather to emphasize broader inflammatory processes of which they are a part[25]. Similarly, we view bFGF and VEGFR-1 as surrogates for other proteins that possess neurotrophic characteristics[26, 27].

We view identified proteins as representatives of other proteins involved in the newborn’s complex inflammatory response[50]. Not all inflammation-related proteins have the same developmental regulation pattern[51], nor do all neurotrophic proteins have the same developmental regulation trajectory[52]. The abilities to complete such tasks as inflammation resolution, brain maturation, and repair of brain damage apparently increases with increasing gestational age[53]. Thus, we rely on the top quartile concentrations of multiple inflammatory and neurotrophic proteins to provide risk information beyond the mere concentration of the individual protein.

### Adjusting for medicaid eligibility

With the evidence that mother’s socioeconomic disadvantage and her other adversities occurs before the onset of inflammation in the fetus and newborn, inflammation is likely in the causal chain between maternal adversities and the child’s neurodevelopmental limitations. In such a situation, adjusting for the maternal adversities will result in “over-adjustment”[54], and possibly diminish the contribution of inflammation to the developmental limitations.

We document that adjusting for Medicaid eligibility minimally alters the relationship between top quartile concentrations of proteins and the risks of low IQ, and separately the risk of a low working memory score among those with an IQ in the normal range. To us, this indicates that the overwhelming contribution of socioeconomic disadvantage to these two “outcomes” is likely via inflammation.

### Integration of immune and neurodevelopment systems

The biological embedding model of early adversity postulates that early adversity can become “embedded” in immune cells, resulting in a "proinflammatory phenotype"[16–18]. Proponents “argue that early-life adversity amplifies crosstalk between peripheral inflammation and neural circuitries”[55]. This argument raises the possibility that we did not measure what they consider most important, “crosstalk between peripheral inflammation and neural circuitries.” We add that possibility to the list of our study’s limitations. This argument also raises the possibility that some of the inflammation we measured continues at a low level, which can be amplified by diminished parenting and other adversities associated with socioeconomic disadvantage.

### Limitations and strengths of this study

We are limited by the relatively small number of proteins measured. Inflammation is a complex and highly inter-related set of processes[56], and we have assessed only a very small part of it. We relied on blood specimens obtained for clinical indications. As the deviance of their blood gas measurements diminished, infants were less likely than their sicker peers to have blood drawn on days 14, 21, and 28. Consequently, selection bias probably occurred to some extent as the number of children for whom we had specimens from both of the late-epoch days tended to be about 2/3 of the number for whom we had specimens from two of the three early-epoch days.

We do not know why low maternal IQ is a less informative indicator/correlate of the biologic consequences of low SES than are Medicaid eligibility and no post-high-school education. One explanation is the reduced power associated with smaller sample size. For example, only 93 mothers satisfied the K-BIT < 85 criterion, whereas more than 300 mothers satisfied the no post-high-school education criterion (N = 353), and the Medicaid eligibility criterion (N = 304) (Fig 1). Another explanation is that the KBIT-2 and the liberal cutoff of 85 we used to define low IQ was not a sensitive measure for assessing the neurocognitive correlates of SES and systemic inflammation in our sample.

This study has sufficient power to identify an odds ratio of 1.4 in the individual-day analyses, 1.5 for multiple days in the early epoch analyses, and 1.8 in the multiple days in the late epoch analyses. Other strengths are the selection of infants based on gestational age, not birth weight[57], prospective collection of all data, and protein data of high quality, and high content validity.

### Conclusion

In this cohort of children born extremely preterm and at high-risk for neurocognitive limitations, maternal socioeconomic disadvantage was modestly associated with an increased inflammatory signal and decreased neurotrophic signal in the blood during the first postnatal month.

## Supporting information

S1 Table Odds ratios and 95% confidence intervals of a top quartile concentration of the protein listed on the left associated with mother’s education (<16 years). (DOCX)

S2 Table : Odds ratios and 95% confidence intervals of a top quartile concentration of the protein listed on the left associated with mother’s IQ (KBIT-2) < 85. (DOCX)

S3 Table Odds ratios (95% confidence intervals) for the 10-year old having an IQ less than 70, and separately, among children whose IQ ≥ 70, having a working memory more than one standard deviation below the expected mean associated with a top-quartile concentration of the protein listed on the left on 2 days during the early epoch. (DOCX)

S4 Table Odds ratios (95% confidence intervals) for the 10-year old having an IQ less than 70, and separately, among children whose IQ ≥ 70, having a working memory more than one standard deviation below the expected mean associated with a top-quartile concentration of the protein listed on the left on 2 days during the early epoch. (DOCX)

S5 Table Odds ratios (95% confidence intervals) for the 10-year old having an IQ less than 70, and separately, among children whose IQ ≥ 70, having a working memory more than one standard deviation below the expected mean associated with a top-quartile concentration of the protein listed on the left on 2 days during the late epoch. (DOCX)

S6 Table Odds ratios (95% confidence intervals) for the 10-year old having an IQ less than 70, and separately, among children whose IQ ≥ 70, having a working memory more than one standard deviation below the expected mean associated with a top-quartile concentration of the protein listed on the left on 2 days during the late epoch. (DOCX)

## Abbreviations

Ang-1: Angiopoietin-1
Ang-2: Angiopoietin-2
BDNF: Brain-Derived Neurotrophic Factor
bFGF: basic Fibroblast Growth Factor
CRP: C-Reactive Protein
DAS-II: Differential Ability Scales–II
EPO: Erythropoietin
ICAM-1: Intercellular Adhesion Molecule -1
IGF-1: Insulin-like growth factor-1
IGFBP-1: Insulin-like growth factor binding protein-1
IL-1β: Interleukin-1 β
IL-6: Interleukin-6
IL-6R: Interleukin-6 Receptor
IL-8: Interleukin-8
IQ: Intelligence quotient
KBIT-2: Kaufman Brief Intelligence Test– 2
MMP-9: Matrix Metalloproteinase-9
MPO: Myeloperoxidase
NT-4: Neurotrophin-4
PIGF: Placenta Growth Factor
RANTES: Regulated upon Activation, Normal T-cell Expressed, and Secreted
SAA: Serum Amyloid A
SES: Socio-economic status
TNF-R1: Tumor Necrosis Factor-α Receptor-1
TNF-R2: Tumor Necrosis Factor-α Receptor-2
TNF-α: Tumor Necrosis Factor-α
TSH: Thyroid-Stimulating Hormone
VCAM-1: Vascular Cell Adhesion Molecule -1
VEGF: Vascular endothelial growth factor
VEGF-R1: Vascular endothelial growth factor Receptor-1
VEGF-R2: Vascular endothelial growth factor Receptor-2
WIAT-III: Wechsler Individual Achievement Test-III
