# Supplementary material for: Socioeconomic status and early blood concentrations of inflammation-related and neurotrophic proteins among extremely preterm newborns
Source: PLoS One. 2019 Mar 26;14(3):e0214154. doi: 10.1371/journal.pone.0214154 (PMC6435168; doi:10.1371/journal.pone.0214154)
Supplement: S1 Table — (DOCX) [file pone.0214154.s001.docx]

**S1 Table**: Odds ratios and 95% confidence intervals of a top quartile concentration of the protein listed on the left associated with mother’s education (<16 years). **Bold** indicates odds ratios significantly > 1.0 (p < 0.05) and ***bold italic*** indicates odds ratios significantly < 1.0 (p < 0.05).

|  | Day 1 | Day 7 | Day 14 | Day 21 | Day 28 |
| --- | --- | --- | --- | --- | --- |
| CRP | 1.0 (0.7, 1.4) | **1.8 (1.2, 2.5)** | 1.2 (0.9, 1.8) | 1.1 (0.7, 1.6) | 0.9 (0.6, 1.4 ) |
| SAA | 1.1 (0.8, 1.5) | **1.5 (1.03, 2.1)** | 1.3 (0.9, 1.9) | 1.0 (0.7, 1.5) | 0.8 (0.6, 1.2) |
| MPO | 0.9 (0.7, 1.3) | 1.3 (0.9, 1.8) | 1.3 (0.9, 1.8) | 0.8 (0.6, 1.3) | 1.0 (0.7, 1.5) |
| IL-1β | 0.9 (0.7, 1.3) | 1.1 (0.8, 1.5) | 1.1 (0.8, 1.6) | 0.9 (0.6, 1.3) | 1.4 (0.9, 2.0) |
| IL-6 | 1.2 (0.8, 1.6) | **1.5 (1.1, 2.2)** | **1.6 (1.1, 2.3)** | 0.9 (0.6, 1.3) | 1.0 (0.7, 1.5) |
| IL-6R | 1.4 (1.00, 2.0) | 1.1 (0.8, 1.5) | 0.9 (0.7, 1.3) | 1.3 (0.9, 1.9) | 1.0 (0.7, 1.5) |
| TNF-α | 1.2 (0.8, 1.6) | **1.7 (1.2, 2.4)** | 1.3 (0.9, 1.8) | 0.9 (0.6, 1.3) | 0.9 (0.6, 1.3) |
| TNF-R1 | 0.9 (0.6, 1.2) | 1.3 (0.9, 1.8) | 1.3 (0.9, 1.9) | 1.3 (0.9, 2.0) | 1.0 (0.7, 1.5) |
| TNF-R2 | 1.2 (0.9, 1.7) | **1.7 (1.2, 2.4)** | **1.9 (1.3, 2.7)** | **1.5 (1.04, 2.3)** | **1.6 (1.04, 2.3)** |
| IL-8 | 1.3 (0.95, 1.9) | 1.3 (0.9, 1.8) | **1.5 (1.1, 2.2)** | 0.9 (0.6, 1.3) | **1.8 (1.2, 2.7)** |
| RANTES | 1.2 (0.9, 1.7) | 1.4 (0.99, 2.0) | 0.9 (0.6, 1.2) | 0.9 (0.6, 1.3) | 1.0 (0.7, 1.5) |
| ICAM-1 | 1.2 (0.8, 1.7) | **1.7 (1.2, 2.4)** | **1.7 (1.2, 2.5)** | 1.0 (0.7, 1.5) | **1.7 (1.1, 2.5)** |
| VCAM-1 | 0.9 (0.7, 1.3) | 1.1 (0.8, 1.6) | 1.1 (0.8, 1.6) | 1.0 (0.6, 1.5) | 0.8 (0.6, 1.2) |
| MMP-9 | 1.1 (0.8, 1.5) | 1.1 (0.8, 1.5) | 1.2 (0.8, 1.7) | 0.8 (0.6, 1.3) | 0.9 (0.6, 1.3) |
| TSH | 1.0 (0.7, 1.4) | **1.7 (1.2, 2.5)** | 1.0 (0.7, 1.4) | 1.0 (0.7, 1.6) | ***0.6 (0.4, 0.9)*** |
| EPO | 1.0 (0.7, 1.3) | 1.4 (0.98, 1.9) | 1.2 (0.9, 1.7) | 0.9 (0.6, 1.3) | 0.7 (0.5, 1.02) |
| NT-4 | 0.8 (0.6, 1.2) | 0.9 (0.7, 1.3) | ***0.6 (0.4, 0.9)*** | 0.8 (0.5, 1.2) | 0.8 (0.6, 1.2) |
| BDNF | 1.2 (0.9, 1.8) | 1.3 (0.9, 1.9) | 0.8 (0.6, 1.1) | 1.0 (0.7, 1.5) | 0.7 (0.5, 1.1) |
| bFGF | 0.7 (0.5, 1.03) | 0.9 (0.6, 1.2) | 0.8 (0.6, 1.2) | 0.8 (0.5, 1.3) | 0.8 (0.6, 1.2) |
| IGF-1 | 0.7 (0.5, 1.1) | 0.9 (0.6, 1.3) | 0.9 (0.7, 1.4) | 0.8 (0.6, 1.3) | 0.8 (0.5, 1.1) |
| IGFBP-1 | 1.0 (0.7, 1.4) | 0.8 (0.6, 1.1) | 1.4 (0.95, 2.0) | 0.9 (0.6, 1.3) | 1.0 (0.7, 1.5) |
| VEGF | 1.1 (0.8, 1.6) | 1.6 (1.1, 2.2) | 1.0 (0.7, 1.4) | 1.0 (0.7, 1.5) | 0.8 (0.5, 1.1) |
| VEGF-R1 | 0.9 (0.7, 1.3) | 0.9 (0.6, 1.2) | ***0.6 (0.5, 0.9)*** | 0.7 (0.5, 1.1) | 0.8 (0.6, 1.2) |
| VEGF-R2 | **1.5 (1.04, 2.1)** | **1.6 (1.1, 2.2)** | **1.7 (1.2, 2.4)** | 1.3 (0.9, 1.9) | 1.1 (0.8, 1.7) |
| PIGF | ***0.7 (0.5, 0.96)*** | 1.0 (0.7, 1.5) | 0.7 (0.5, 1.1) | 0.9 (0.7, 1.4) | 0.8 (0.6, 1.2) |
| Ang-1 | 1.4 (0.97, 2.0) | 1.0 (0.7, 1.5) | ***0.7 (0.5, 0.96)*** | 0.9 (0.6, 1.4) | 0.7 (0.5, 1.1) |
| Ang-2 | 1.0 (0.7, 1.5) | 1.3 (0.9, 1.8) | 1.0 (0.7, 1.5) | 1.0 (0.7, 1.5) | 1.0 (0.7, 1.5) |
