# Supplementary material for: Socioeconomic status and early blood concentrations of inflammation-related and neurotrophic proteins among extremely preterm newborns
Source: PLoS One. 2019 Mar 26;14(3):e0214154. doi: 10.1371/journal.pone.0214154 (PMC6435168; doi:10.1371/journal.pone.0214154)
Supplement: S4 Table — (DOCX) [file pone.0214154.s004.docx]

**S4 Table.** Odds ratios (95% confidence intervals) for the 10-year old having an IQ less than 70, and separately, among children whose IQ ≥ 70, having a working memory more than one standard deviation below the expected mean associated with a top-quartile concentration of the protein listed on the left on 2 days during the **early** epoch. In one set of analyses adjustment is made for mother’s IQ (KBIT-2) < 85 at the time of delivery, while in another, such adjustment is not made although in both situations adjustment is made for gestational age category and birth weight Z-score < -1. **Bold** indicates odds ratios significantly > 1 (p < 0.05) and ***bold italic*** indicates odds ratios significantly < 1 (p < 0.05).

|  |  |  | | Working memory Z-score ≤ -1 | |
| --- | --- | --- | --- | --- | --- |
|  |  | IQ < 70 | | when IQ ≥ 70 | |
|  | KBIT-2 | Adjusted for KBIT-2 < 85 | | Adjusted for KBIT-2 < 85 | |
|  | < 85 * | No | Yes | No | Yes |
| CRP | 1.1 (0.7, 1.9) | 1.5 (0.95, 2.5) | 1.5 (0.9, 2.5) | 1.3 (0.8, 2.0) | 1.3 (0.8, 2.0) |
| SAA | 1.2 (0.7, 2.1) | 1.3 (0.8, 2.2) | 1.3 (0.8, 2.2) | 1.2 (0.7, 1.9) | 1.1 (0.7, 1.8) |
| MPO | 1.2 (0.7, 2.0) | 1.0 (0.6, 1.7) | 1.0 (0.6, 1.7) | 0.8 (0.5, 1.2) | 0.7 (0.5, 1.2) |
| IL-1β | 0.8 (0.5, 1.6) | 1.4 (0.8, 2.3) | 1.5 (0.9, 2.4) | 1.1 (0.7, 1.7) | 1.1 (0.7, 1.7) |
| IL-6 | **1.9 (1.1, 3.2)** | **1.8 (1.1, 3.0)** | 1.7 (1.00, 2.8) | 1.1 (0.7, 1.9) | 1.1 (0.7, 1.8) |
| IL-6R | **1.7 (1.1, 2.9)** | ***0.5 (0.3, 0.97)*** | ***0.5 (0.2, 0.9)*** | 0.9 (0.6, 1.4) | 0.9 (0.6, 1.4) |
| TNF-α | 1.4 (0.8, 2.4) | 1.5 (0.9, 2.5) | 1.5 (0.9, 2.4) | **2.0 (1.3, 3.1)** | **2.0 (1.3, 3.1)** |
| TNF-R1 | 0.8 (0.5, 1.5) | 1.2 (0.7, 2.1) | 1.3 (0.8, 2.1) | 1.2 (0.8, 1.9) | 1.2 (0.8, 2.0) |
| TNF-R2 | 1.3 (0.8, 2.3) | 1.3 (0.8, 2.2) | 1.3 (0.8, 2.1) | 1.1 (0.7, 1.7) | 1.1 (0.7, 1.7) |
| IL-8 | 1.0 (0.6, 1.8) | **1.9 (1.2, 3.1)** | **1.9 (1.2, 3.2)** | **1.7 (1.1, 2.7)** | **1.7 (1.1, 2.7)** |
| RANTES | 1.5 (0.9, 2.5) | 0.8 (0.4, 1.4) | 0.7 (0.4, 1.3) | 0.9 (0.6, 1.4) | 0.9 (0.5, 1.4) |
| ICAM-1 | 1.2 (0.7, 2.1) | **2.0 (1.3, 3.2)** | **2.0 (1.2, 3.2)** | 1.5 (0.9, 2.3) | 1.5 (0.9, 2.3) |
| VCAM-1 | 1.4 (0.8, 2.4) | 1.2 (0.7, 2.0) | 1.1 (0.7, 1.9) | 1.1 (0.7, 1.7) | 1.1 (0.7, 1.7) |
| MMP-9 | 1.0 (0.5, 1.8) | 0.7 (0.4, 1.4) | 0.7 (0.4, 1.4) | 1.1 (0.7, 1.7) | 1.1 (0.6, 1.7) |
| TSH | 1.2 (0.7, 2.0) | 1.0 (0.6, 1.7) | 1.0 (0.6, 1.7) | 1.1 (0.7, 1.8) | 1.1 (0.7, 1.7) |
| EPO | 1.0 (0.5, 1.7) | 1.6 (0.9, 2.5) | 1.6 (0.95, 2.6) | **1.7 (1.1, 2.6)** | **1.7 (1.1, 2.6)** |
| NT-4 | 0.9 (0.5, 1.6) | 1.3 (0.8, 2.1) | 1.3 (0.8, 2.2) | 1.3 (0.9, 2.0) | 1.3 (0.8, 2.1) |
| BDNF | 1.5 (0.9, 2.5) | 0.6 (0.3, 1.1) | ***0.5 (0.3, 0.99)*** | 0.9 (0.6, 1.4) | 0.8 (0.5, 1.3) |
| bFGF | 0.8 (0.4, 1.6) | 1.0 (0.6, 1.8) | 1.1 (0.6, 1.9) | 1.0 (0.6, 1.7) | 1.1 (0.6, 1.7) |
| IGF-1 | 0.8 (0.5, 1.6) | 1.0 (0.6, 1.8) | 1.0 (0.6, 1.8) | 0.7 (0.4, 1.1) | 0.7 (0.4, 1.1) |
| IGFBP-1 | 0.9 (0.5, 1.7) | 1.1 (0.6, 1.9) | 1.1 (0.6, 2.0) | 1.1 (0.7, 1.8) | 1.1 (0.7, 1.9) |
| VEGF | 0.8 (0.5, 1.5) | 0.7 (0.4, 1.2) | 0.7 (0.4, 1.2) | 1.0 (0.7, 1.6) | 1.0 (0.7, 1.6) |
| VEGF-R1 | ***0.4 (0.1, 0.9)*** | 0.7 (0.4, 1.2) | 0.8 (0.4, 1.4) | 1.0 (0.6, 1.6) | 1.0 (0.7, 1.7) |
| VEGF-R2 | 0.9 (0.5, 1.6) | 1.1 (0.6, 1.7) | 1.0 (0.6, 1.7) | 1.2 (0.8, 1.9) | 1.2 (0.8, 1.9) |
| PIGF | 0.6 (0.3, 1.3) | 0.6 (0.3, 1.3) | 0.7 (0.3, 1.4) | 1.1 (0.7, 1.8) | 1.2 (0.7, 1.9) |
| Ang-1 | 1.5 (0.8, 2.1) | ***0.5 (0.2, 0.96)*** | ***0.4 (0.2, 0.9)*** | 0.6 (0.4, 1.04) | ***0.6 (0.4, 0.99***) |
| Ang-2 | 0.7 (0.4, 1.4) | 1.0 (0.5, 1.7) | 1.0 (0.6, 1.8) | 1.1 (0.7, 1.9) | 1.2 (0.7, 1.9) |

***** Repeated from Table 3
