# Supplementary material for: Socioeconomic status and early blood concentrations of inflammation-related and neurotrophic proteins among extremely preterm newborns
Source: PLoS One. 2019 Mar 26;14(3):e0214154. doi: 10.1371/journal.pone.0214154 (PMC6435168; doi:10.1371/journal.pone.0214154)
Supplement: S5 Table — (DOCX) [file pone.0214154.s005.docx]

**S5 Table.** Odds ratios (95% confidence intervals) for the 10-year old having an IQ less than 70, and separately, among children whose IQ ≥ 70, having a working memory more than one standard deviation below the expected mean associated with a top-quartile concentration of the protein listed on the left on 2 days during the **late** epoch. In one set of analyses adjustment is made for mother’s education < 16 years at the time of delivery, while in another, such adjustment is not made although in both situations adjustment is made for gestational age category and birth weight Z-score < -1. **Bold** indicates odds ratios significantly > 1 (p < 0.05) and ***bold italic*** indicates odds ratios significantly < 1 (p < 0.05).

|  |  |  | | Working memory Z-score ≤ -1 | |
| --- | --- | --- | --- | --- | --- |
|  |  | IQ < 70 | | when IQ ≥ 70 | |
|  | Education | Adjusted for education < 16 yr | | Adjusted for education < 16 yr | |
|  | < 16 yr * | No | Yes | No | Yes |
| CRP | 1.0 (0.6, 1.7) | **2.0 (1.03, 3.7)** | **2.0 (1.03, 3.8)** | 1.5 (0.8, 2.9) | 1.5 (0.9, 3.0) |
| SAA | 0.6 (0.3, 1.1) | 1.9 (0.9, 3.9) | 2.1 (0.99, 4.3) | 1.3 (0.6, 2.7) | 1.4 (0.6, 2.9) |
| MPO | 0.8 (0.5, 1.4) | 0.8 (0.4, 1.7) | 0,8 (0.4, 1.7) | 1.3 (0.7, 2.3) | 1.3 (0.7, 2.5) |
| IL-1β | 1.3 (0.7, 2.4) | 1.0 (0.5, 2.1) | 1.0 (0.5, 2.0) | 1.2 (0.6, 2.2) | 1.1 (0.6, 2.1) |
| IL-6 | 0.9 (0.5, 1.5) | 1.8 (0.96, 3.5) | 1.9 (0.98, 3.6) | 1.5 (0.8, 2.8) | 1.6 (0.9, 3.0) |
| IL-6R | 1.6 (0.9, 2.8) | 0.9 (0.4, 1.8) | 0.8 (0.4, 1.7) | 0.9 (0.5, 1.7) | 0.8 (0.4, 1.7) |
| TNF-α | 0.7 (0.5, 1.2) | 1.6 (0.9, 2.8) | 1.7 (0.9, 3.0) | 1.0 (0.6, 1.8) | 1.0 (0.6, 1.9) |
| TNF-R1 | 0.9 (0.5, 1.6) | 1.1 (0.5, 2.3) | 1.1 (0.5, 2.3) | 0.7 (0.3, 1.5) | 0.7 (0.3, 1.5) |
| TNF-R2 | **1.9 (1.1, 3.5)** | 1.2 (0.6, 2.3) | 1.1 (0.6, 2.2) | 1.3 (0.7, 2.4) | 1.2 (0.6, 2.2) |
| IL-8 | **1.8 (1.02, 3.0)** | **2.7 (1.5, 4.8)** | **2.6 (1.4, 4.6)** | **2.1 (1.1, 3.8)** | **1.9 (1.05, 3.5)** |
| RANTES | 1.0 (0.6, 1.7) | 1.6 (0.9, 3.1) | 1.6 (0.8, 3.1) | 0.6 (0.3, 1.3) | 0.6 (0.3, 1.2) |
| ICAM-1 | **1.7 (1.01, 2.8)** | **2.4 (1.3, 4.2)** | **2.3 (1.3, 4.0)** | **1.8 (1.01, 3.2)** | 1.7 (0.96, 3.1) |
| VCAM-1 | 0.9 (0.5, 1.6) | 0.9 (0.4, 1.8) | 0.9 (0.4, 1.9) | 0.8 (0.4, 1.5) | 0.8 (0.4, 1.5) |
| MMP-9 | 1.3 (0.7, 2.4) | 1.2 (0.6, 2.5) | 1.2 (0.5, 2.5) | 0.9 (0.4, 1.9) | 0.9 (0.4, 1.9) |
| TSH | 0.7 (0.4, 1.1) | **2.0 (1.1, 3.5)** | **2.1 (1.1, 3.8)** | 0.8 (0.4, 1.5) | 0.9 (0.4, 1.7) |
| EPO | ***0.6 (0.3, 0.99)*** | 1.1 (0.6, 2.2) | 1.2 (0.6, 2.5) | 0.9 (0.5, 1.8) | 1.0 (0.5, 1.9) |
| NT-4 | 0.7 (0.4, 1.2) | 1.5 (0.9, 2.9) | 1.6 (0.9, 3.0) | 1.1 (0.6, 2.2) | 1.2 (0.6, 2.3) |
| BDNF | 0.7 (0.4, 1.2) | 0.8 (0.4, 1.7) | 0.8 (0.3, 1.7) | 0.6 (0.3, 1.2) | 0.6 (0.3, 1.3) |
| bFGF | ***0.5 (0.3, 0.9)*** | 0.9 (0.4, 1.9) | 1.0 (0.5, 2.2) | 0.8 (0.4, 1.6) | 0.9 (0.4, 1.7) |
| IGF-1 | 0.8 (0.5, 1.3) | ***0.4 (0.2, 0.9)*** | ***0.4 (0.2, 0.9)*** | 1.0 (0.6, 1.8) | 1.0 (0.6, 1.8) |
| IGFBP-1 | 1.0 (0.6, 1.9) | **2.1 (1.01, 4.2)** | **2.1 (1.01, 4.2)** | 1.0 (0.5, 2.2) | 1.0 (0.5, 2.2) |
| VEGF | 0.6 (0.4, 1.1) | 0.9 (0.5, 1.9) | 1.0 (0.5, 2.0) | 1.1 (0.6, 2.0) | 1.2 (0.6, 2.2) |
| VEGF-R1 | 0.9 (0.5, 1.5) | 0.6 (0.3, 1.4) | 0.6 (0.3, 1.5) | 1.5 (0.8, 2.7) | 1.5 (0.8, 2.7) |
| VEGF-R2 | 1.7 (0.9, 2.9) | 1.7 (0.9, 3.2) | 1.6 (0.9, 3.1) | 1.2 (0.6, 2.3) | 1.2 (0.6, 2.1) |
| PIGF | 0.6 (0.3, 1.1) | 0.8 (0.3, 1.9) | 0.9 (0.4, 2.0) | 0.6 (0.3, 1.3) | 0.6 (0.3, 1.4) |
| Ang-1 | 0.6 (0.4, 1.04) | 0.7 (0.3, 1.6) | 0.8 (0.4, 1.7) | 0.7 (0.4, 1.3) | 0.7 (0.4, 1.4) |
| Ang-2 | 1.2 (0.8, 2.1) | **1.9 (1.1, 3.3)** | **1.9 (1.03, 3.2)** | 0.9 (0.5, 1.6) | 0.8 (0.5, 1.6) |

***** Repeated from Table 4
